# Informative HPV testing after conization and its impact on time-varying estimates: a GAMM-based cohort study

**DOI:** 10.3389/fpubh.2026.1808122

**Published:** 2026-04-29

**Authors:** Jie Zhou, Jian hong Liao, Lin Jie Su, Yan Chen, Hong bo Hu

**Affiliations:** 1 Department of Gynecology, Yuebei People's Hospital Affiliated to Shantou University Medical College, Shaoguan, Guangdong, China; 2 Department of Gynecology, First People's Hospital of Zhaoqing, Zhaoqing, Guangdong, China

**Keywords:** HPV, HPV infection, HPV single infection, multiple types of HPV infections, time-varying pattern (TVP)

## Abstract

**Objectives:**

To characterize post-conization time-varying HPV positivity trajectories in a real-world setting with irregular follow-up, and to assess whether the association between baseline infection multiplicity (single vs. multiple) and postoperative HPV positivity is robust to testing intensity and potential informative visiting.

**Methods:**

In this retrospective cohort of 872 women who underwent conization and completed ≥1 postoperative HPV test, we modeled HPV positivity (yes/no) over continuous months since conization using generalized additive mixed models with subject-specific random effects. Time-varying between-group differences were evaluated using a group-specific time-varying parameter. To address irregular follow-up and potential informative visiting, we performed prespecified sensitivity analyses by (i) restricting to women with ≥3 postoperative HPV tests and (ii) additionally adjusting for testing intensity (number of HPV tests during follow-up). Models adjusted for age, menopausal status, admission type, health insurance type, margin status, preoperative cytology, gravidity/parity, and postoperative hysterectomy.

**Results:**

In the overall cohort, postoperative HPV positivity decreased over time in both groups (per 4 months: single infection OR = 0.66, 95% CI 0.63–0.70; multiple infection OR = 0.71, 95% CI 0.65–0.78; both *P* < 0.0001). Multiple infection was associated with a persistently higher time-varying trajectory (OR = 1.22, 95% CI 1.12–1.32; *P* < 0.0001), with differences concentrated in the early-to-mid follow-up period. Findings were consistent in sensitivity analyses restricting to women with ≥3 tests and after adjustment for testing intensity.

**Conclusions:**

Post-conization HPV testing occurs under irregular, potentially informative follow-up in real-world practice. Accounting for testing intensity, the association between baseline multiple infection and less favorable time-varying postoperative trajectories remained robust, supporting risk-stratified surveillance while highlighting the need to explicitly consider follow-up patterns when estimating postoperative HPV dynamics.

## 1 Introduction

Cervical conization is widely regarded as an effective diagnostic and therapeutic approach for cervical precancerous lesions, enabling removal of diseased tissue and reduction of progression risk (1, 2). However, a proportion of patients still experience persistent or recurrent human papillomavirus (HPV) positivity after surgery, which increases the risk of lesion recurrence and the burden of postoperative surveillance (3). Previous studies have suggested that postoperative infection type, margin status, and pathological findings are associated with the persistence of high-risk HPV infection (4, 5). Nevertheless, existing evidence has largely focused on HPV status at several fixed postoperative follow-up time points, with relatively limited follow-up durations (6). As a result, it remains unclear how these factors shape the longitudinal, time-varying trajectory of postoperative HPV positivity—rather than outcomes at a single visit—under routine care.

Multiple HPV infections may further complicate postoperative risk assessment and surveillance planning (7). Several studies suggest that multiple infections are associated with lower HPV clearance and less favorable postoperative HPV outcomes (8), yet evidence regarding their association with high-grade squamous intraepithelial lesions (HSIL) has been inconsistent (9, 10). These discrepancies suggest that the effect of multiple infections may depend on genotype composition, as high-risk HPV types such as HPV16, 52, and 58 differ in oncogenic potential and persistence characteristics (11). Therefore, evaluating longitudinal postoperative HPV dynamics by baseline infection multiplicity within genotype-restricted contexts may help clarify whether the impact of multiple infection is uniform or varies by genotype background.

A major challenge for longitudinal postoperative HPV research is that real-world follow-up is often irregular, with unequal test numbers and variable visit intervals across patients. Moreover, definitions of “transient vs. persistent infection” and “HPV clearance” are not standardized across studies (12, 13), which complicates cross-study comparisons and may bias inferences when follow-up intensity is related to clinical indications (i.e., informative visiting). Conventional analytical approaches may be inadequate. To address these challenges, generalized additive mixed models (GAMMs) allow for the analysis of repeated binary outcomes within a mixed-effects framework and flexibly model time effects using smooth functions, making them well suited for longitudinal data with irregular follow-up intervals and unbalanced observations (14). Accordingly, the present study applies GAMMs to characterize the non-linear time-varying trajectory of postoperative HPV positivity after conization and to quantify differences between baseline single and multiple infections, including genotype-restricted analyses. By additionally evaluating robustness to follow-up patterns and testing intensity, our findings aim to inform risk-stratified surveillance and optimization of postoperative HPV testing strategies.

## 2 Methods

### 2.1 Study design and cohort characteristics

This study was designed as a retrospective cohort study. Patients who underwent their first cervical conization at Yuebei People's Hospital, Shaoguan, Guangdong Province, China, between January 1, 2019, and December 30, 2024, were included. Cervical conization was performed according to clinical indications, including: high-grade squamous intraepithelial lesion (HSIL) indicated by thinprep cytologic test (TCT) with negative or unsatisfactory colposcopic assessment; marked discordance between cytology and colposcopy-guided biopsy pathology; or HSIL confirmed by colposcopy-guided biopsy.

**Figure 1:**
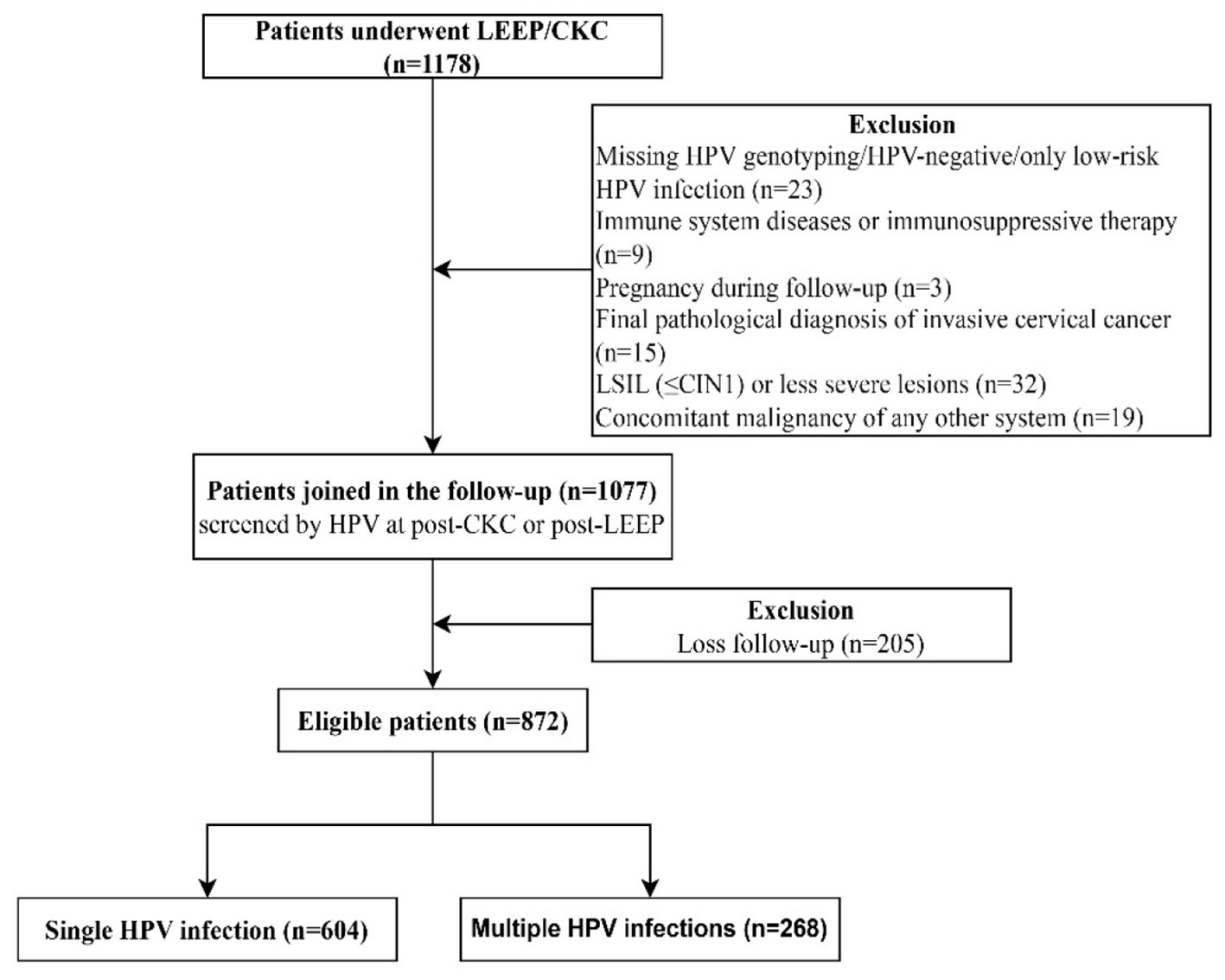
Flow chart of study population.

The follow-up inclusion criteria were as follows: (1) patients meeting indications for cervical conization and undergoing first-time conization; (2) availability of complete and reliable clinical data and follow-up records; (3) presence of one or more high-risk HPV infections prior to surgery; (4) completion of at least one postoperative HPV test during follow-up; and (5) a final pathological diagnosis of HSIL (cervical intraepithelial neoplasia grade 2 or 3, CIN2/3), defined as the highest-grade lesion identified from either biopsy or conization specimens. Exclusion criteria included: (1) missing preoperative HPV genotyping data; (2) negative preoperative HPV status or infection with low-risk HPV types only; (3) immune system–related diseases or receipt of immunosuppressive therapy; (4) pregnancy during the follow-up period; (5) absence of postoperative HPV follow-up with loss to follow-up; (6) a final pathological diagnosis of invasive cervical carcinoma or low-grade squamous intraepithelial lesion (LSIL) or lower ( ≤ cervical intraepithelial neoplasia grade 2, CIN1); and (7) the presence of any other concurrent systemic malignancy (Figure 1).

Clinical data were extracted from the electronic medical record system and included age, menopausal status, HPV genotypes, conization method, hospitalization pattern, medical insurance type, margin status, preoperative TCT results, residual or recurrent disease, and performance of hysterectomy after conization. HPV genotyping was conducted using a National Medical Products Administration–approved assay capable of detecting 23 HPV genotypes, including 17 high-risk and 6 low-risk types. TCT was performed using liquid-based cytology preparation and Papanicolaou staining, and results were reported according to the 2014 Bethesda System, independently interpreted by two senior physicians. Pathological diagnoses were reviewed and confirmed by two senior pathologists.Conization procedures included loop electrosurgical excision procedure (LEEP) and cold knife conization (CKC), and were performed in standard operating rooms by attending gynecologists or physicians with higher qualifications. Margin status was assessed by two senior pathologists; a positive surgical margin was defined as the presence of CIN2 or higher-grade lesions within 1 mm of the resection margin. The analytical population of this study was restricted to patients with a final pathological diagnosis of HSIL (CIN2/3). This study was approved by the Ethics Committee of Yuebei People's Hospital (Approval No. YBSKY-2023-135-002). All data were anonymized, and the requirement for written informed consent was waived.

### 2.2 Exposure definition and genotype-restricted subcohorts

To evaluate differences related to infection patterns and genotype-specific effects, infection status was defined based on preoperative HPV genotyping. Single HPV infection was defined as detection of only one HPV genotype, whereas multiple HPV infection was defined as detection of two or more HPV genotypes. Single vs. multiple infection was defined according to the preoperative HPV genotyping results and was treated as a baseline exposure (prognostic marker), without dynamic updating during follow-up. Based on prior evidence that HPV genotypes differ in persistence and recurrence risk, we *a priori* conducted genotype-restricted analyses to examine whether the association between baseline infection multiplicity (single vs. multiple infection) and postoperative HPV positivity trajectories varied by genotype background. Specifically, we evaluated HPV16+/HPV18- (baseline HPV16+/HPV18-), HPV52+/HPV16/18-, and HPV58+/HPV16/18- subcohorts (baseline HPV52+/HPV16/18- and HPV58+/HPV16/18-, respectively), which were chosen before model fitting because these genotypes are prevalent in our setting and have documented clinical relevance (15). Multiple infections were defined using baseline (pre-conization) HPV genotyping: detection of one HPV genotype was classified as single infection, and detection of ≥2 genotypes as multiple infection. This exposure was treated as baseline-fixed and was not reclassified according to genotype changes during follow-up. The purpose of these analyses was effect modification or heterogeneity assessment, rather than discovery of genotype-specific associations. The overall cohort analysis was the primary analysis; genotype-restricted analyses were secondary and interpreted as supportive evidence of heterogeneity.

### 2.3 Follow-up and outcomes

The time points and results of each postoperative HPV retesting were recorded for all participants, with follow-up extending through December 2024. The primary outcome of this study was defined as HPV positivity for any genotype at each post-operative follow-up visit (i.e., regardless of whether the detected genotype was the same as the baseline type). Therefore, our findings reflect the temporal dynamics of overall HPV positivity and cannot distinguish persistent infection, new infection, or reactivated infection. The timing of post-operative HPV retesting was mainly scheduled according to the routine clinical follow-up pathway at our center; however, the actual retesting time points could be adjusted because of concurrent cytological abnormalities, suspicious colposcopic findings, or differences in patient adherence. The post-operative follow-up workflow at our center is shown in Supplementary Figure 5. This figure is intended to summarize the routine follow-up pathway and its possible deviations at our center, and does not imply that all patients were re-examined according to a strictly fixed schedule. Time was defined as the elapsed time (in months) from conization to each HPV test, and each test contributed one longitudinal observation. Because postoperative HPV testing was not performed at fixed intervals, we used the exact follow-up time for each test without enforcing equally spaced measurements. Within-subject correlation arising from repeated tests was accounted for using subject-specific random effects in the generalized additive mixed model (GAMM). To facilitate interpretation, time effects were reported on a per-4-month scale (i.e., model-estimated effects were scaled to represent changes over 4 months), while the model itself was fitted using continuous time in months.

## 3 Statistical analysis

Categorical variables were summarized as counts (percentages), and continuous variables were expressed as mean ± standard deviation or median (interquartile range), as appropriate. Generalized additive mixed models (GAMMs) were applied to model the overall nonlinear temporal trends within a mixed-effects framework. Random effects were incorporated to account for within-subject correlations due to repeated measurements and unbalanced follow-up, making this approach particularly suitable for real-world longitudinal data. After multivariable adjustment, time-varying effect parameters were used to quantify changes in between-group effects over time, allowing differences in HPV clearance trajectories to be expressed in an interpretable manner and providing a statistical foundation for subsequent risk stratification model development. The models specified HPV status as a binary outcome with a logit link function, and subject-specific random effects were included to handle repeated measures. Using generalized additive mixed models (mgcv::gamm, binomial logit), time (DAY/120; ~4 months per unit) was modeled with cubic regression splines (bs = “cr”, *k* = 6), with group-specific smooths by baseline infection multiplicity and a subject-specific random intercept. Two models were constructed: Model I, unadjusted; and Model II, adjusted for age, menopausal status, conization method, hospitalization pattern, medical insurance type, margin status, preoperative TCT results, residual or recurrent disease, and postoperative hysterectomy (consistent with table footnotes). The number of post-operative HPV tests was included as a covariate to approximate overall follow-up intensity; however, this approach cannot fully eliminate bias arising from clinically indicated visiting. Between-group differences were evaluated using group-by-time time-varying interaction terms (TVP_group) and were reported separately for the overall cohort and genotype-restricted subcohorts. Time-segmented analyses were conducted by fitting models separately before and after the prespecified thresholds. Results were presented as odds ratios (ORs) with 95% confidence intervals (CIs). TVP_single and TVP_multiple represent the relative change in the odds of HPV positivity per 4 months in the single- and multiple-infection groups, respectively; TVP_group represents the relative ratio of these time-related changes in odds between the two groups and is used to quantify between-group differences in temporal trajectories. The segmented thresholds were determined by jointly considering the follow-up distribution, curve shape, and the amount of information available in the later follow-up period for each cohort. These thresholds were primarily used for descriptive segmented analyses and therefore represent an exploratory, partly data-driven specification. A two-sided *P* value <0.05 was considered statistically significant. All statistical analyses were performed using Empower (R) and R software (version 4.2.2).

## 4 Sensitivity analyses

To assess the robustness of the primary GAMM estimates under irregular follow-up and potential informative visiting, we conducted the following prespecified sensitivity analyses: (1) Participants with relatively regular follow-up were included, requiring at least three HPV tests after surgery. (2) The main model was additionally adjusted for the total number of HPV tests during follow-up (Supplementary Figures 4, 6).

## 5 Result

### 5.1 Patient characteristics

This retrospective cohort study included 872 patients who completed at least one postoperative HPV test following cervical conization. Baseline clinical characteristics and the distribution of key covariates are presented in Table 1, including age, menopausal status, HPV16/18 infection status, hospitalization type, medical insurance type, conization pathology, margin status, and postoperative hysterectomy. In addition, the proportions of single and multiple HPV infections as well as the composition of genotype-restricted subcohorts are summarized. The median (IQR) number of post-conization HPV tests was 3 (2–5). As a supplementary descriptive analysis, we further calculated the clearance proportions of key baseline high-risk HPV types within 12, 24, and 36 months after surgery to aid interpretation of the primary outcome defined as “positivity for any HPV genotype” (Supplementary Table 5). There were no statistically significant differences between the baseline single- and multiple-infection groups in total follow-up duration, interval between adjacent tests, or number of post-operative HPV tests (Supplementary Table 6), suggesting no apparent association between baseline multiple infection and overall follow-up intensity. The cumulative incidence is presented in Supplementary Table 8.

**Table 1:** Baseline characteristics of patients after conization.

Characteristics	Single infection (*N* = 604)	Multiple infection (*N* = 268)	*P*-value
Age (years), mean (SD)	43.61 (9.58)	43.44 (11.86)	0.824
Number of postoperative HPV tests, Median (Q1-Q3)	3.00 (2.00–5.00)	3.00 (2.00–6.00)	0.867
gravidity, mean (SD)	3.28 (1.77)	3.37 (1.71)	0.475
parity, mean (SD)	1.85 (1.01)	2.14 (1.30)	<0.001
Hysterectomy, *n* (%)			
No	440 (72.85%)	198 (73.88%)	0.751
Yes	164 (27.15%)	70 (26.12%)	
Type of surgery, *n* (%)			
Loop electrosurgical excision procedure	545 (90.23%)	245 (91.42%)	0.46
Cold knife conization	59 (9.77%)	23 (8.58%)	
Menopause, *n* (%)			
Premenopausal	474 (78.48%)	198 (73.88%)	0.136
Postmenopausal	130 (21.52%)	70 (26.12%)	
Type of hospitalization, *n* (%)			
Ambulatory surgery	133 (22.02%)	54 (20.15%)	0.535
Inpatient surgery	471 (77.98%)	214 (79.85%)	
Type of Insurance, *n* (%)			
Resident medical insurance	307 (50.82%)	135 (50.37%)	0.585
Employee medical insurance	260 (43.05%)	112 (41.80%)	
Inter-regional medical insurance	37 (6.13%)	21 (7.83%)	
Pre-conization TCT, *n* (%)			
NILM	127 (21.03%)	64 (23.88%)	0.867
ASC-US	123 (20.37%)	50 (18.66%)	
LSIL	133 (22.01%)	62 (23.13%)	
HSIL	221 (36.59%)	92 (34.33%)	
Recurrence or persistence, *n* (%)			
No	498 (82.45%)	190 (70.90%)	<0.001
Yes	106 (17.55%)	78 (29.10%)	
**95% CI**	**13.52%, 19.41%**	**21.86%, 32.15%**	
Positive margin status, *n* (%)			
No	456 (75.50%)	204 (76.12%)	0.843
Yes	148 (24.50%)	64 (23.88%)	
HPV16, *n* (%)			
No	356 (58.94%)	143 (53.36%)	0.124
Yes	248 (41.06%)	125 (46.64%)	
HPV18, *n* (%)			
No	545 (90.15%)	232 (86.63%)	0.182
Yes	59 (9.85%)	36 (13.37%)	
HPV52, *n* (%)			
No	491 (81.29%)	177 (66.04%)	<0.001
Yes	113 (18.71%)	91 (33.96%)	
HPV58, *n* (%)			
No	515 (85.23%)	198 (73.83%)	<0.001
Yes	89 (14.77%)	70 (26.17%)	

Data are expressed as the mean ± SD or percentage. Definitions of the acronims: NILM, Negative for Intraepithelial Lesion or Malignancy; ASC-US, Atypical Squamous Cells of Undetermined Significance; LSIL, Low-grade Squamous Intraepithelial Lesion; HSIL, High-grade Squamous Intraepithelial Lesion. Bold values indicate statistically significant *P*-values (*P* < 0.05).

### 5.2 Overall cohort: time-varying postoperative HPV positivity in single vs. multiple infections

**Figure 2:**
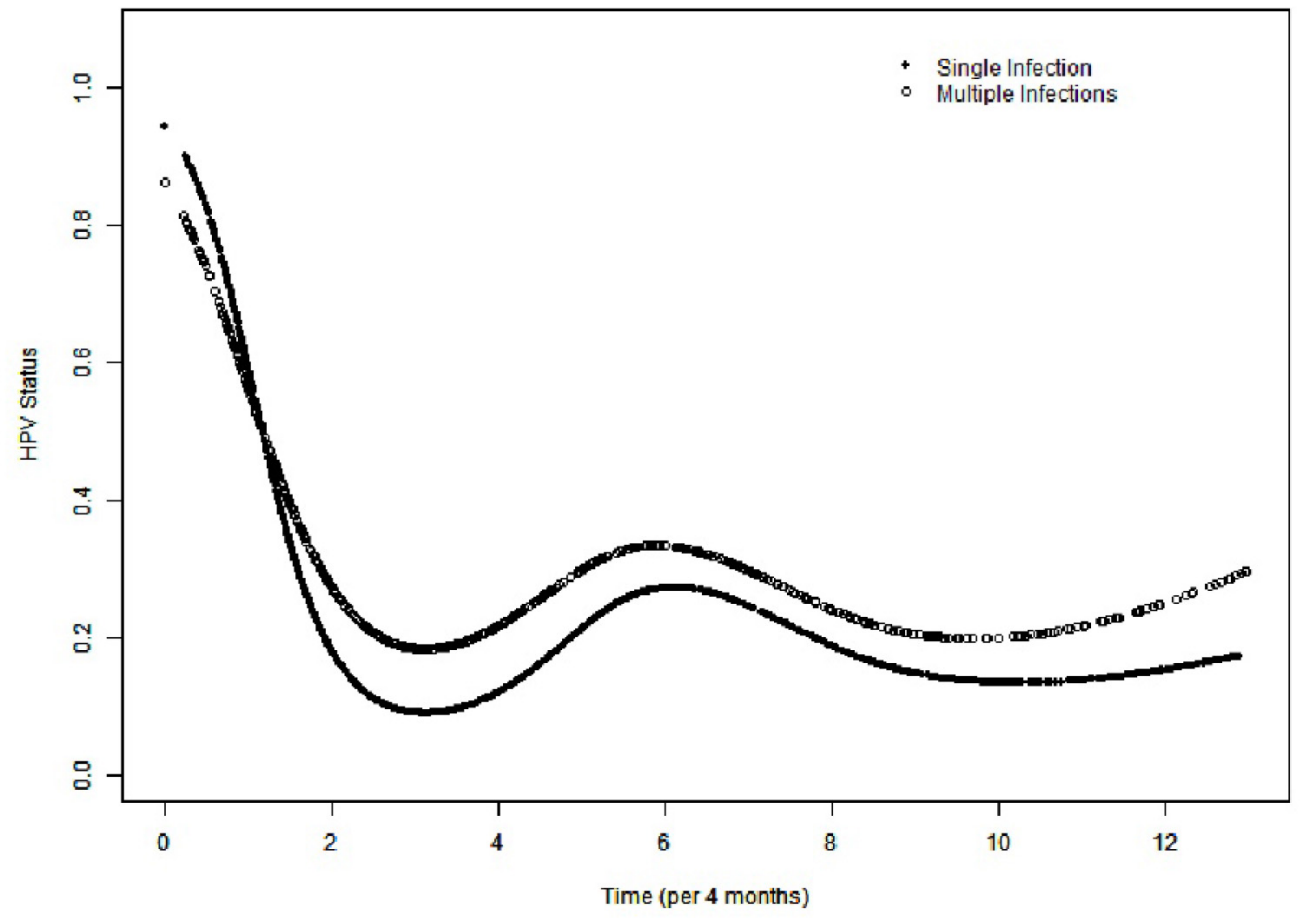
Compares the time-varying pattern (TVP) of HPV status following conization between thesingle HPV infection and multiple HPV infection.

Model-estimated HPV positivity declined over time in both groups, with an early rapid decrease followed by a slower decline (Figure 2). In GAMM analyses (Table 2), the odds of HPV positivity decreased significantly throughout follow-up in both the single- and multiple-infection groups (per 4 months; Model II: single infection OR = 0.66, 95% CI: 0.63–0.70; multiple infection OR = 0.71, 95% CI: 0.65–0.78; both *P* < 0.0001). The time-varying between-group difference was significant (OR = 1.22, 95% CI: 1.12–1.32; *P* < 0.0001). In segmented analyses (Supplementary Table 1), the effect was primarily observed within the first 40 months (Model II: OR = 1.24, 95% CI: 1.09–1.41; *P* = 0.0011). Results were consistent in sensitivity analyses restricted to women with ≥3 tests and after additional adjustment for testing intensity (Supplementary Figure 4). The effective degrees of freedom were 4.95 (single infection) and 4.78 (multiple infection), with both smooth terms highly significant (*P* < 0.001), supporting non-linear postoperative trajectories. Model-predicted probabilities of HPV positivity in the two groups at 12, 24, and 36 months after surgery (or the corresponding relative ratios) with 95% confidence intervals (Supplementary Table 7). As an exploratory analysis, Kaplan–Meier curves for CIN2+ persistence/recurrence stratified by baseline single and multiple infection status are shown in (Supplementary Figure 7).

**Table 2:** Time-varying changes in HPV infection after conization grouped by number of HPV infections using generalized additive mixed models.

Outcome	Model I		Model II	
	OR(95% CI)	*p* value	OR(95% CI)	*p* value
Times (increase per 4 months)				
TVP^single^	0.67 (0.64, 0.71)	<0.0001	0.66 (0.63, 0.70)	<0.0001
TVP^multiple^	0.72 (0.66, 0.78)	<0.0001	0.71 (0.65, 0.78)	<0.0001
TVP: group (multiple infections)	1.21 (1.12, 1.30)	<0.0001	1.22 (1.12, 1.32)	<0.0001

Model I: non-adjusted. Model II: adjusted for age, menopausal status, type of hospitalization, type of health insurance, margin status, pre-conization TCT, gravidity, parity, and postoperative hysterectomy.

### 5.3 HPV16+/HPV18–subcohort: time-varying postoperative HPV positivity in single vs. multiple infections

In the HPV16-positive/HPV18-negative subcohort, the group-specific trajectories gradually converged and crossed at approximately 40 months (Supplementary Figure 1). GAMM results (Table 3) showed significant declines in HPV positivity over time in both groups (per 4 months; Model II: single infection OR = 0.70, 95% CI: 0.65–0.76; multiple infection OR = 0.72, 95% CI: 0.64–0.80; both *P* < 0.0001). The overall was not significant (OR = 1.07, 95% CI: 0.95–1.20; *P* = 0.2497). However, segmented analyses (Supplementary Table 2) indicated a significant time-varying group difference within the first 40 months (Model II: OR = 1.45, 95% CI: 1.21–1.74; *P* < 0.0001).

**Table 3:** Temporal dynamics of post-conization HPV infection stratified by single vs. multiple HPV16 infection^†^.

Outcome	Model I		Model II	
	OR(95% CI)	*p* value	OR(95% CI)	*p* value
All times (increase per 4 months)				
TVP ^HPV16 Single infection^	0.71 (0.66, 0.76)	<0.0001	0.70 (0.65, 0.76)	<0.0001
TVP ^HPV16 Multiple infections^	0.72 (0.65, 0.79)	<0.0001	0.72 (0.64, 0.80)	<0.0001
TVP: group ^HPV16 Multiple infections^	1.07 (0.96, 1.18)	0.2110	1.07 (0.95, 1.20)	0.2497

Model I: non-adjusted. Model II: adjusted for age, menopausal status, type of hospitalization, type of health insurance, margin status, pre-conization TCT, gravidity, parity, and postoperative hysterectomy. ^†^HPV16-positive and HPV18-negative.

### 5.4 HPV52+/HPV16/18– subcohort: postoperative HPV temporal dynamics in single vs. multiple infections

In the HPV52-positive/HPV16/18-negative Subcohort, the multiple-infection group maintained higher model-estimated HPV positivity over time (Supplementary Figure 2). GAMM analyses (Table 4) showed significant temporal declines in both groups (Model II: single infection OR = 0.68, 95% CI: 0.61–0.77; multiple infection OR = 0.79, 95% CI: 0.70–0.89). The overall remained significant (OR = 1.30, 95% CI: 1.11–1.51; *P* = 0.0008). Segmented analyses (Supplementary Table 3) further showed that the time-varying group difference remained significant within 32 months of follow-up (Model II: OR = 1.23, 95% CI: 1.02–1.48; *P* = 0.0273).

**Table 4:** Temporal dynamics of post-conization HPV infection stratified by single vs. multiple HPV52 infection^†^.

Outcome	Model I		Model II	
	OR (95% CI)	*p* value	OR (95% CI)	*p* value
All times (increase per 4 months)				
TVP ^HPV52 Single infection^	0.70 (0.62–0.78)	<0.0001	0.68 (0.61, 0.77)	<0.0001
TVP ^HPV52 Multiple infections^	0.82 (0.71, 0.93)	0.0026	0.79 (0.70, 0.89)	0.0001
TVP: group ^HPV52 Multiple infections^	1.31 (1.13, 1.52)	0.0004	1.30 (1.11, 1.51)	0.0008

Model I: non-adjusted. Model II: adjusted for age, menopausal status, type of hospitalization, type of health insurance, margin status, pre-conization TCT, gravidity, parity, and postoperative hysterectomy. ^†^Baseline HPV52-positive without HPV16/18 co-infection (HPV16-negative, HPV18-negative).

### 5.5 HPV58+/HPV16/18– subcohort: postoperative HPV temporal dynamics in single vs. multiple infections

Among patients who were HPV58-positive without HPV16/18 co-infection, Supplementary Figure 3 indicates that the multiple infection group generally maintained higher model-estimated HPV positivity levels. GAMM results (Table 5) showed that HPV positivity declined significantly over time in both groups (per 4 months: Model II, single infection OR = 0.60, 95% CI 0.48–0.75; multiple infection OR = 0.76, 95% CI: 0.64–0.91). The overall adjusted remained statistically significant (OR = 1.30, 95% CI: 1.06–1.61; *P* = 0.0136). Segmented analysis (Supplementary Table 4) revealed a more pronounced decline in HPV positivity within the first 20 months in both groups (Model II, single infection OR = 0.27, 95% CI 0.15–0.46; multiple infection OR = 0.24, 95% CI: 0.12–0.51). However, the group-by-time interaction did not reach statistical significance in either the <20-month or ≥20-month periods (OR = 1.51, 95% CI: 0.87–2.64; *P* = 0.1440; and OR = 1.24, 95% CI: 0.56–2.75; *P* = 0.5932, respectively).

**Table 5:** Temporal dynamics of post-conization HPV infection stratified by single vs. multiple HPV58 infection^†^.

Outcome	Model I		Model II	
	OR (95% CI)	*p* value	OR (95% CI)	*p* value
All times (increase per 4 months)				
TVP ^HPV58 Single infection^	0.68 (0.60, 0.78)	<0.0001	0.60 (0.48, 0.75)	<0.0001
TVP ^HPV58 Multiple infections^	0.80 (0.70, 0.91)	0.0006	0.76 (0.64, 0.91)	0.0027
TVP:group ^HPV58 Multiple infections^	1.24 (1.05, 1.47)	0.0126	1.30 (1.06, 1.61)	0.0136

Model I: non-adjusted. Model II: adjusted for age, menopausal status, type of hospitalization, type of health insurance, margin status, pre-conization TCT, gravidity, parity, and postoperative hysterectomy. ^†^Baseline HPV58-positive without HPV16/18 co-infection (HPV16-negative, HPV18-negative).

## 6 Discussion

In this real-world post-conization surveillance cohort, we used generalized additive mixed models (GAMMs) to characterize the non-linear time-varying trajectory of postoperative HPV positivity and to assess whether baseline infection multiplicity (single vs. multiple) was associated with differential postoperative trajectories. Overall, HPV positivity declined over time with a pronounced non-linear pattern. Importantly, women with baseline multiple infections consistently exhibited a less favorable time-varying postoperative trajectory than those with single infections, and this association persisted after multivariable adjustment for key clinical factors (including age, menopausal status, margin status, preoperative cytology, gravidity/parity, and postoperative hysterectomy). Given that postoperative HPV testing is often conducted at irregular intervals and may be influenced by clinical indications, we further evaluated robustness to follow-up patterns by restricting analyses to women with ≥3 postoperative tests and by additionally accounting for testing intensity; the direction of associations remained consistent, supporting that the observed trajectory differences were not solely driven by differential testing behavior. The between-group divergence was most evident in the early-to-mid follow-up window (<40 months), highlighting a clinically relevant period for risk-informed surveillance. Genotype-restricted analyses additionally suggested heterogeneity by baseline genotype background, with comparatively more stable differences in the HPV52+/HPV16/18– (and HPV58+/HPV16/18–) subcohorts, indicating that postoperative risk stratification may benefit from jointly considering infection multiplicity and genotype context.

Previous studies have demonstrated that cervical conization can substantially reduce HPV positivity rates (13), and that HPV test results at specific postoperative time points (e.g., 6, 12, or 24 months) are associated with recurrence risk (16, 17). However, most existing evidence is based on fixed follow-up time points or linear assumptions, which limits the ability to provide direct evidence for optimizing follow-up intervals across populations with different risk profiles (18). Current risk-based management frameworks likewise emphasize that surveillance intensity should be matched to individual risk levels (19, 20). Given that postoperative HPV status may dynamically shift between negative and positive states, we conceptualized postoperative HPV positivity as a continuous-time process. This approach not only quantified the nonlinear temporal evolution of overall risk but also revealed time-window–specific features of the association between multiple infection and postoperative HPV positivity, thereby offering potential evidence to support more refined postoperative surveillance strategies.

Multiple HPV infection was associated with a higher risk of postoperative HPV positivity, and this elevated risk persisted over time compared with single infection. This phenomenon may be related to the more complex viral ecology and host microenvironment associated with the coexistence of multiple HPV genotypes (9). First, multiple infection often reflects a higher viral load or a broader extent of infected epithelial fields (21–23). Even after conization removes the main lesion, residual infected cells or a “field effect” may remain (24, 25), delaying viral clearance. Second, co-infection with different genotypes may influence host viral clearance efficiency through differences in immune evasion properties and inter-genotype competition or synergy (26–29). Third, multiple infection may serve as a marker of weaker host immune control or higher exposure risk, and may therefore be accompanied by persistent HPV positivity and an increased tendency toward lesion persistence or recurrence (30, 31). In the present study, we also exploratorily observed a higher proportion of residual or recurrent disease in the multiple infection group (Table 1), which is directionally consistent with these hypotheses. Nevertheless, these mechanisms require further validation through studies incorporating genotype-specific dynamics, viral load measurements, and immunological indicators. Notably, we found that between-group differences were primarily evident during the early postoperative follow-up period, suggesting that multiple infection may particularly indicate the need for intensified early surveillance, whereas long-term differences warrant confirmation in larger cohorts.

In genotype-restricted analyses, we observed heterogeneity in the association between multiple infection and postoperative HPV positivity across genotypes. Specifically, in the HPV52+/16/18– subcohort, multiple infection was consistently associated with a higher risk of postoperative HPV positivity throughout follow-up. In contrast, in the HPV16+/18– subcohort, between-group differences were mainly confined to the early follow-up window, suggesting that the impact of multiple infection on postoperative HPV risk in the HPV16 background may be time-dependent. In the HPV58+/16/18– subcohort, although overall estimates suggested between-group differences, statistical significance was not achieved within segmented time windows, possibly due to reduced sample size after stratification. These genotype-specific findings are directionally consistent with prior epidemiological evidence: HPV16, 52, and 58 are among the most prevalent high-risk HPV types in Chinese women, and multiple infections involving these genotypes are closely associated with CIN2+ lesions (32, 33). A recent study from southern China reported relatively faster clearance of HPV16 and slower clearance of HPV52, which may contribute to a greater difficulty in achieving negativity for certain genotypes (34). Another study similarly identified HPV58 as an important predictor of poor treatment response, particularly in the context of multiple infection, further supporting the notion that multiple-infected patients are more likely to experience delayed clearance (35). Collectively, these findings support incorporating infection multiplicity—together with genotype background—into postoperative risk stratification, with particular attention to its impact on HPV positivity during early critical follow-up windows.

Taken together, the nonlinear pattern of postoperative HPV positivity suggests that uniform, fixed-interval surveillance strategies may not be equally appropriate across different risk groups. Preoperative multiple infection, especially when considered alongside genotype background, may serve as a potentia risk stratification indicator to support more conservative HPV (± cytology) follow-up strategies. However, such risk-adaptive approaches require validation through multicenter prospective studies and cost-effectiveness analyses.

This study has several limitations. First, as a single-center retrospective cohort study, its external generalizability is limited. Second, the primary outcome was defined as post-operative positivity for any HPV genotype, which precluded differentiation among persistent infection, new infection, and reactivated infection. Accordingly, the findings should be interpreted as associations between baseline infection pattern and subsequent HPV temporal dynamics, rather than causal effects. In addition, because HPV status and the visiting process were not jointly modeled, residual bias due to clinically indicated repeat testing may remain. Furthermore, incomplete medical record data limited our ability to obtain detailed type-specific longitudinal follow-up information and to fully account for potential confounders, including HPV vaccination, smoking, sexual behavior, and immunosuppression. Finally, the bimodal-like patterns observed in some early post-operative trajectory plots likely reflect sparse early data, boundary effects in spline fitting, and heterogeneity in visit timing, rather than true virological processes. Further multicenter prospective studies are warranted to validate these findings.

In summary, postoperative HPV positivity following cervical conization exhibits a nonlinear temporal pattern. Preoperative multiple HPV infection is associated with an increased risk of persistent postoperative HPV positivity, with this association being most pronounced during early follow-up and demonstrating genotype-specific heterogeneity. These findings quantitatively elucidate the time-varying association between infection multiplicity and postoperative HPV clearance trajectories, As candidate indicators for the validation of future risk stratification follow-up strategies.

## Data Availability

The raw data supporting the conclusions of this article will be made available by the authors, without undue reservation.
