# Supplementary figures and images for: Informative HPV testing after conization and its impact on time-varying estimates: a GAMM-based cohort study
Source: Front Public Health. 2026 Apr 29;14:1808122. doi: 10.3389/fpubh.2026.1808122 (PMC13168214; doi:10.3389/fpubh.2026.1808122)

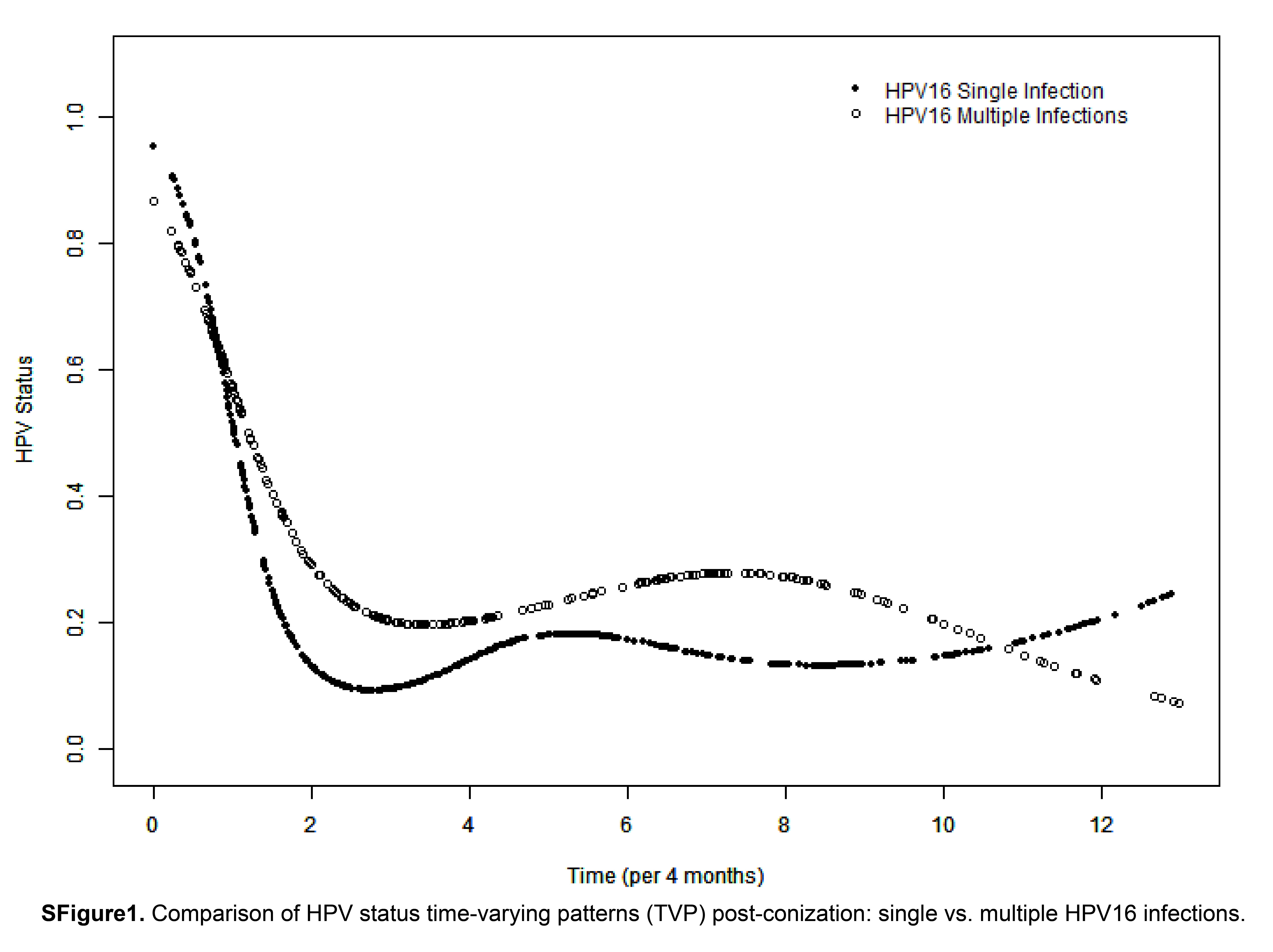

Supplement: Supplementary file 1 [file Image_1.jpeg]

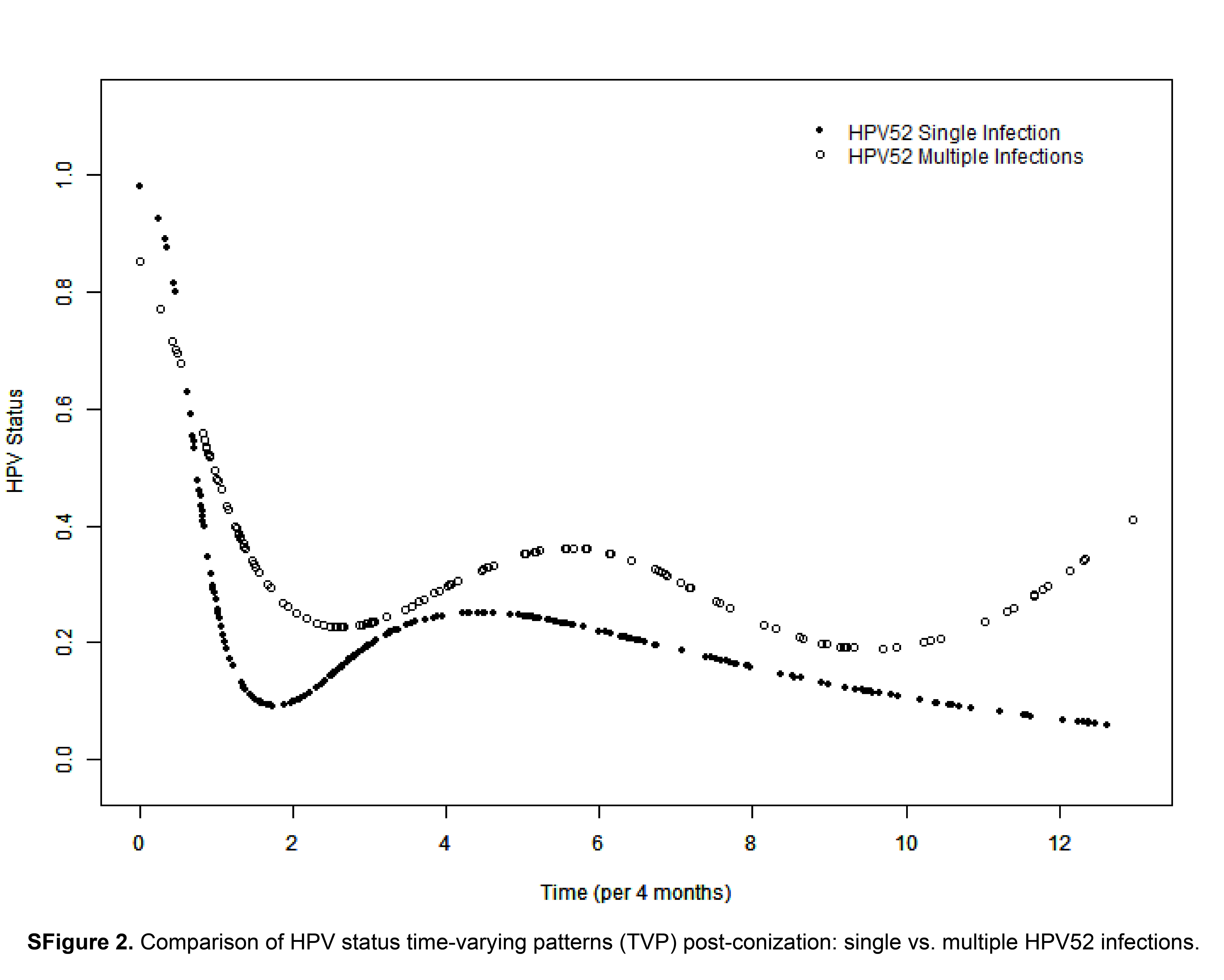

Supplement: Supplementary file 2 [file Image_2.jpeg]

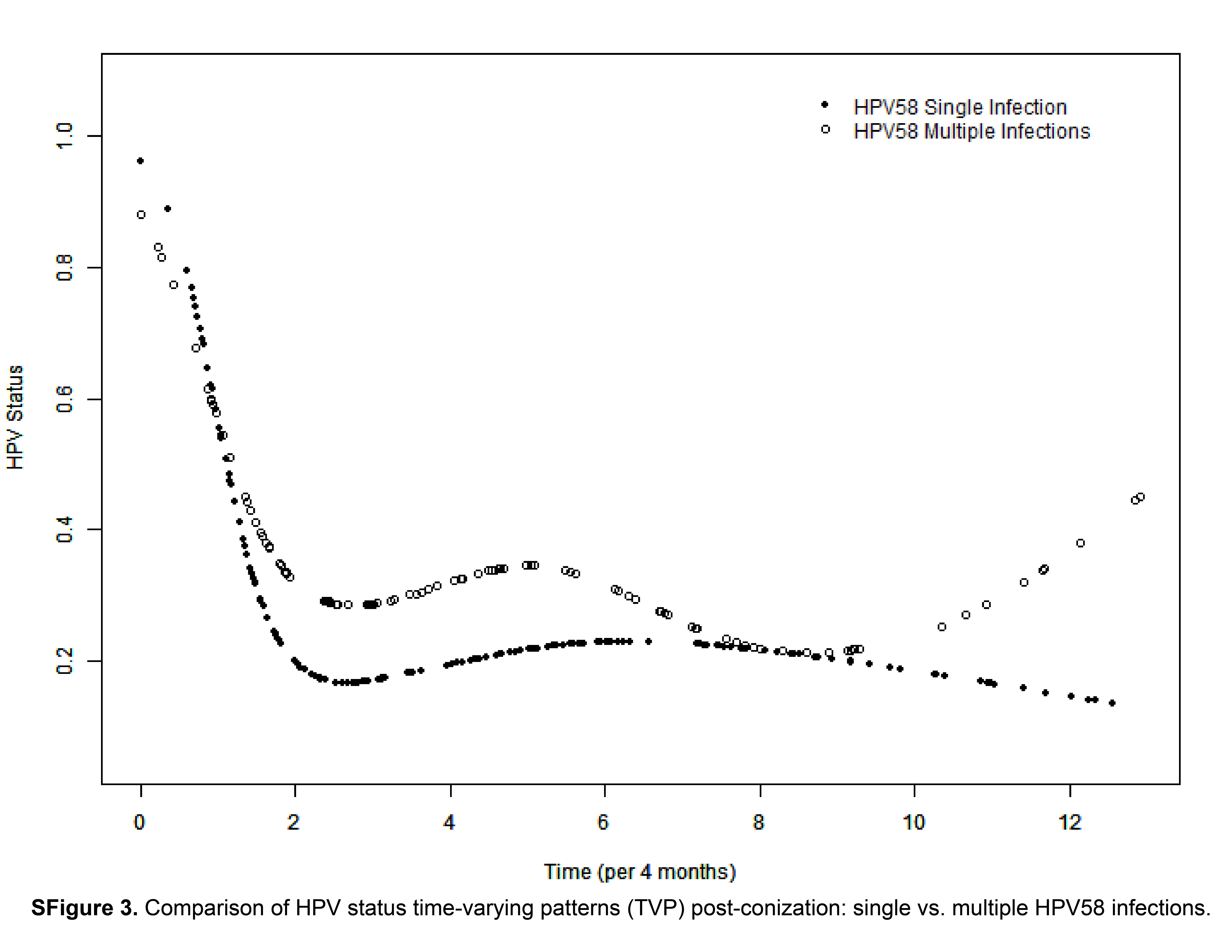

Supplement: Supplementary file 3 [file Image_3.jpeg]

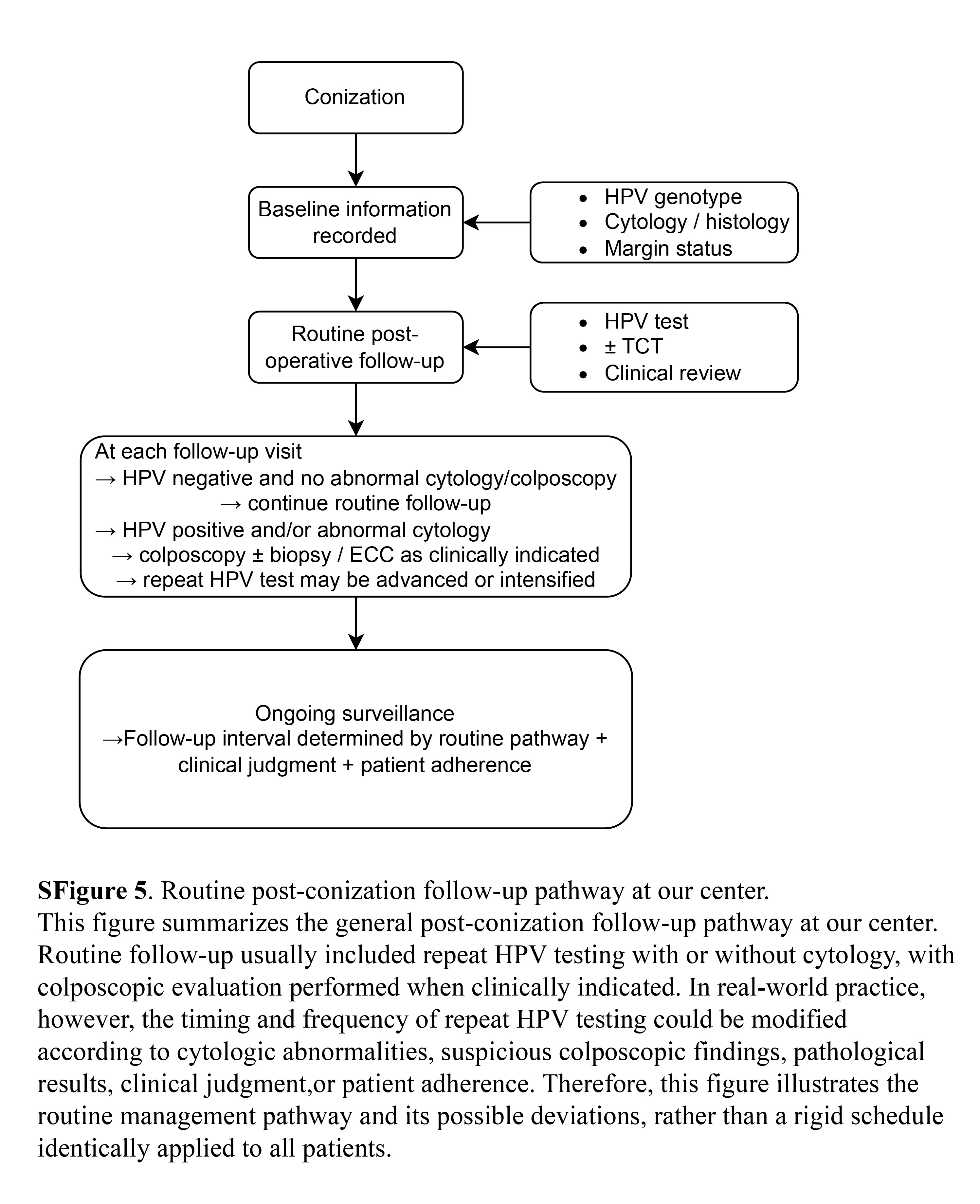

Supplement: Supplementary file 5 [file Image_5.jpeg]

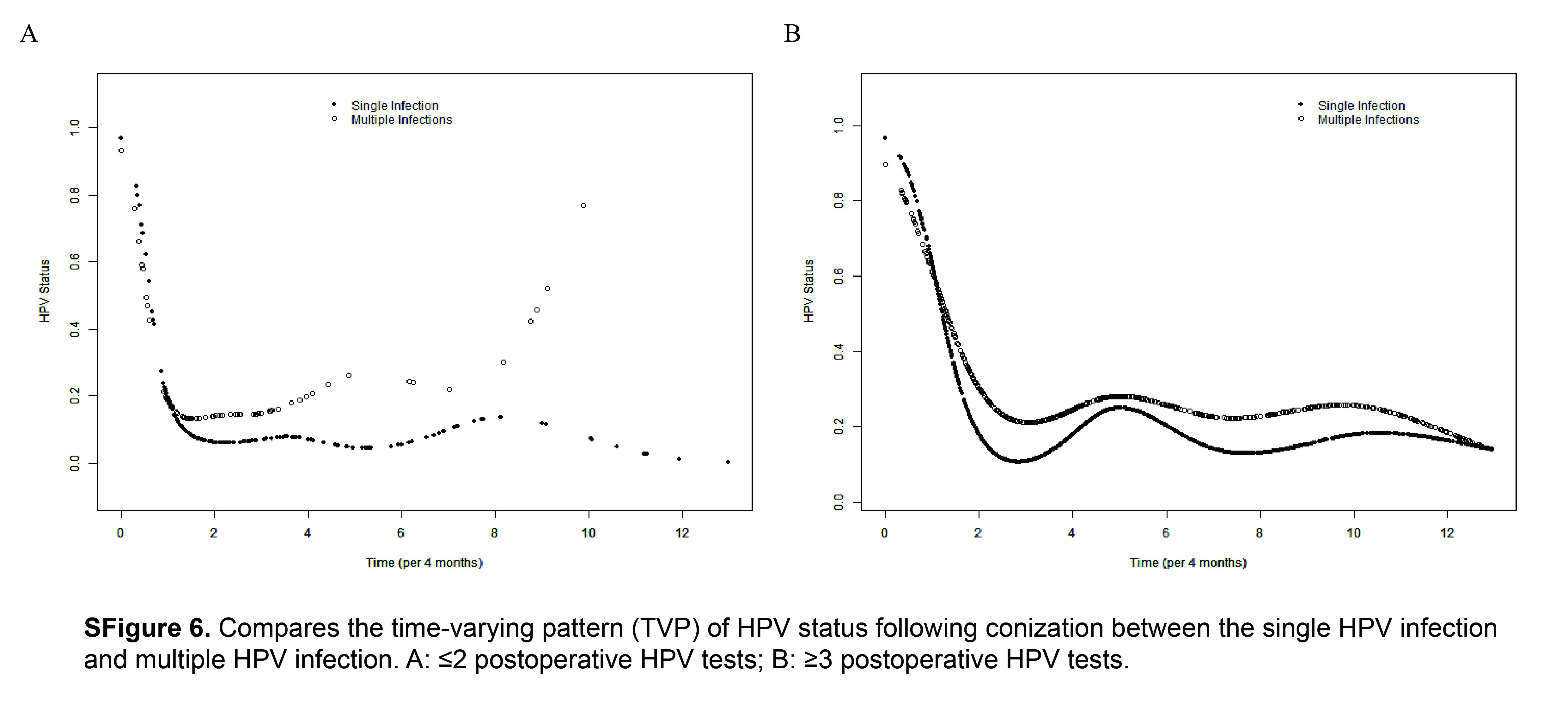

Supplement: Supplementary file 6 [file Image_6.jpeg]

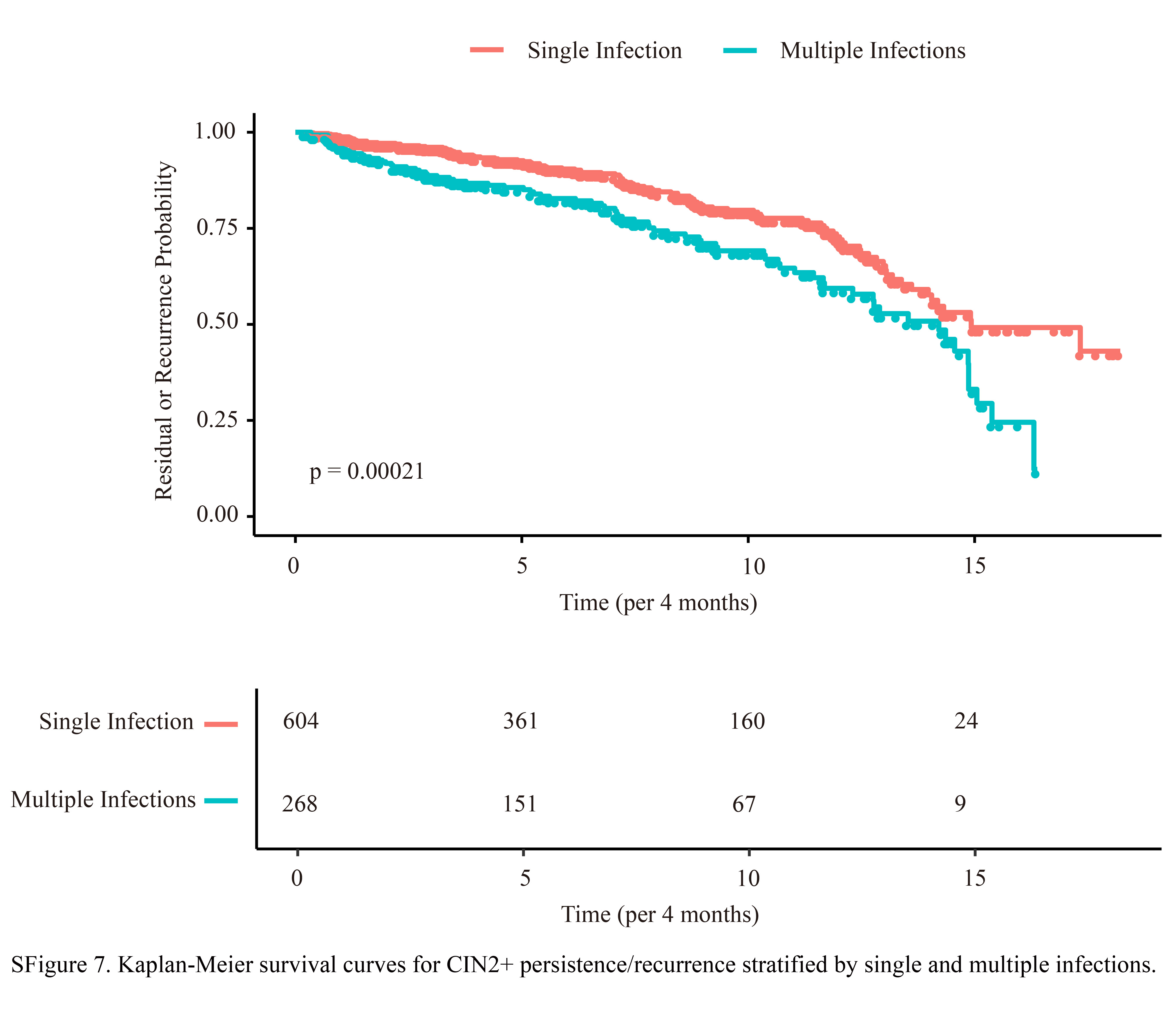

Supplement: Supplementary file 7 [file Image_7.jpeg]
