# Supplementary material for: Informative HPV testing after conization and its impact on time-varying estimates: a GAMM-based cohort study
Source: Front Public Health. 2026 Apr 29;14:1808122. doi: 10.3389/fpubh.2026.1808122 (PMC13168214; doi:10.3389/fpubh.2026.1808122)
Supplement: Supplementary file 9 [file Supplementary_file_1.docx]

| **STable 1** Stage-specific temporal dynamics of post-conization HPV infection according to HPV infection multiplicity: results from a generalized additive mixed model | | | | | |
| --- | --- | --- | --- | --- | --- |
| Outcome | Model I | |  | Model II | |
|  | OR (95%CI) | p value |  | OR (95%CI) | p value |
| **Time ≥ 40 months (increase per 4 months)** |  |  |  |  |  |
| TVP ^Single infection^ | 0.61 (0.57,0.64) | <0.0001 |  | 0.57 (0.52,0.63) | <0.0001 |
| TVP^Multiple infections^ | 0.68 (0.62,0.73) | <0.0001 |  | 0.68 (0.60,0.76) | <0.0001 |
| TVP:group^(Multiple infections)^ | 1.22 (1.13,1.32) | <0.0001 |  | 1.24 (1.09,1.41) | 0.0011 |
|  |  |  |  |  |  |
| **Time < 40months (increase per 4 months)** |  |  |  |  |  |
| TVP ^Single infection^ | 0.91 (0.50,1.66) | 0.7613 |  | / | / |
| TVP ^Multiple infections^ | 0.87 (0.87,0.88) | <0.0001 |  | 0.91  (0.59,1.39) | 0.6505 |
| TVP:group^(Multiple infections)^ | 0.94 (0.39,2.27) | 0.8881 |  | 1.07  (0.58,1.98) | 0.8346 |
| Model I: Non-adjusted.Model II: Adjusted for age, menopausal status, type of hospitalization, type of health insurance, margin status, pre-conization TCT, gravidity, parity, and postoperative hysterectomy. | | | | | |

| **STable2** Stage-specific temporal dynamics of post-conization HPV infection stratified by single vs. multiple HPV16 infection† | | | | | |
| --- | --- | --- | --- | --- | --- |
| Outcome | Model I | |  | Model II | |
|  | OR(95%CI) | p value |  | OR(95%CI) | p value |
| **Time ≥ 40 months (increase per 4 months)** |  |  |  |  |  |
| TVP ^HPV16 Single infection^ | 0.54  (0.47,0.61) | <0.0001 |  | 0.52  (0.45,0.60) | <0.0001 |
| TVP ^HPV16 Multiple infections^ | 0.67 (0.59,0.77) | <0.0001 |  | 0.68  (0.60,0.78) | <0.0001 |
| TVP:group ^HPV16 Multiple infections^ | 1.32 (1.14,1.53) | 0.0003 |  | 1.45  (1.21,1.74) | <0.0001 |
|  |  |  |  |  |  |
| **Time < 40months (increase per 4 months)** |  |  |  |  |  |
| TVP ^HPV16 Single infection^ | 1.40  (1.39,1.40) | <0.0001 |  | **/** | **/** |
| TVP ^HPV16 Multiple infections^ | 0.62 (0.35,1.10) | 0.1030 |  | **/** | **/** |
| TVP:group ^HPV16 Multiple infections^ | 0.01 (0.00,2.45) | 0.1052 |  | **/** | **/** |
| Model I: Non-adjusted.Model II: Adjusted for age, menopausal status, type of hospitalization, type of health insurance, margin status, pre-conization TCT, gravidity, parity, and postoperative hysterectomy. † HPV16-positive and HPV18-negative. | | | | | |

| **Stable 3** Stage-specific temporal dynamics of post-conization HPV infection stratified by single vs. multiple HPV52 infection † | | | | | |
| --- | --- | --- | --- | --- | --- |
| Outcome | Model I | |  | Model II | |
|  | OR(95%CI) | p value |  | OR(95%CI) | p value |
| **Time ≤ 32months (increase per 4 months)** |  |  |  |  |  |
| TVP ^HPV52 Single infection^ | 0.66  (0.57,0.76) | <0.0001 |  | 0.67  (0.58,0.77) | <0.0001 |
| TVP ^HPV52 Multiple infections^ | 0.76  (0.64,0.90) | 0.0016 |  | 0.74  (0.63,0.87) | 0.0003 |
| TVP:group ^HPV52 Multiple infections^ | 1.28  (1.08,1.53) | 0.0044 |  | 1.23  (1.02,1.48) | 0.0273 |
|  |  |  |  |  |  |
| **Time > 32months (increase per 4 months)** |  |  |  |  |  |
| TVP ^HPV52 Single infection^ | 0.98  (0.01,86.50) | 0.9913 |  | 0.92  (0.42,2.01) | 0.8302 |
| TVP ^HPV52 Multiple infections^ | 1.16  (0.09,15.14) | 0.9076 |  | 1.24  (0.55,2.76) | 0.6039 |
| TVP:group ^HPV52 Multiple infections^ | 1.19  (0.01,130.51) | 0.9415 |  | 1.96  (0.67,5.78) | 0.2216 |
| Model I: Non-adjusted.Model II: Adjusted for age, menopausal status, type of hospitalization, type of health insurance, margin status, pre-conization TCT, gravidity, parity, and postoperative hysterectomy. † Baseline HPV52-positive without HPV16/18 co-infection (HPV16-negative, HPV18-negative). | | | | | |

| **STable 4** Stage-specific temporal dynamics of post-conization HPV infection stratified by single vs. multiple HPV58 infection † | | | | | |
| --- | --- | --- | --- | --- | --- |
| Outcome | Model I | |  | Model II | |
|  | OR(95%CI) | p value |  | OR(95%CI) | p value |
| **Time<20 months (increase per 4 months)** |  |  |  |  |  |
| TVP ^HPV58 Single infection^ | 0.20  (0.10,0.40) | <0.0001 |  | 0.27  (0.15,0.46) | <0.0001 |
| TVP ^HPV58 Multiple infections^ | 0.21  (0.06,0.70) | 0.0109 |  | 0.24  (0.12,0.51) | 0.0002 |
| TVP:group ^HPV58 Multiple infections^ | 1.84  (0.873.87) | 0.1085 |  | 1.51  (0.87,2.64） | 0.1440 |
|  |  |  |  |  |  |
| **Time ≥ 20 months (increase per 4 months)** |  |  |  |  |  |
| TVP ^HPV58 Single infection^ | 0.65  (0.39,1.09) | 0.1041 |  | 0.61  (0.32,1.18) | 0.1421 |
| TVP ^HPV58 Multiple infections^ | 0.91  (0.68,1.23) | 0.5413 |  | 1.16  (0.43,3.10) | 0.7717 |
| TVP:group ^HPV58 Multiple infections^ | 1.14  (0.62,2.09) | 0.6797 |  | 1.24  (0.56,2.75) | 0.5932 |
| Model I: Non-adjusted.Model II: Adjusted for age, menopausal status, type of hospitalization, type of health insurance, margin status, pre-conization TCT, gravidity, parity, and postoperative hysterectomy. † Baseline HPV58-positive without HPV16/18 co-infection (HPV16-negative, HPV18-negative). | | | | | |

| **STable 5.** Overall post-conization HPV clearance at 12, 24, and 36 months among patients stratified by baseline high-risk HPV type | | | |  |
| --- | --- | --- | --- | --- |
| Baseline type | 12-month clearance rate | 24-month clearance rate | 36-month clearance rate |  |
| Any hrHPV | 512/872 (58.71%) | 636/872 (73.00%) | 670/872 (76.90%) |  |
| HPV16 | 207/373 (55.49%) | 265/373 (71.04%) | 285/373 (76.41%) |  |
| HPV52 | 136/204 (66.66%) | 157/204 (76.96%) | 164/204(80.39%) |  |
| HPV58 | 88/159 (55.34%) | 107/159 (67.29%) | 114/159 (71.69%) |  |
| Note: Baseline type was determined according to the record with follow-up time equal to 0 days. Clearance was defined as having at least one follow-up visit with overall HPV negativity (HPV positivity status = 0) within 12, 24, or 36 months after conization. The denominator in each row represents the total number of patients with that baseline high-risk HPV type. Because the current longitudinal dataset records overall HPV positive/negative status at each follow-up visit, rather than type-specific HPV results at each visit, this table reflects overall HPV clearance in populations defined by baseline HPV type, rather than strict persistence/clearance of the same baseline genotype. Therefore, persistent infection, new infection, and reactivated infection could not be distinguished. | | | |  |
|  |  |  |  |  |
|  |  |  |  |  |
|  |  |  |  |  |
|  |  |  |  |  |
|  |  |  |  |  |

| **STable 6.** Follow-up process and post-operative HPV testing intensity stratified by baseline infection pattern | | | | | |  |  |
| --- | --- | --- | --- | --- | --- | --- | --- |
| **Variable** | | **Single infection group (n=604)** | | **Multiple infection group (n=268)** | | **P-value** |  |
| Total follow-up duration (days), median (IQR) | | 736.50 (337.25–1250.25) | | 728.00 (288.50–1184.25) | | 0.14 |  |
| Time to first post-operative follow-up (days), median (IQR) | | 159.00 (146.00–173.00) | | 148.00 (127.00–162.00) | | 0.24 |  |
| Interval between consecutive post-operative HPV tests (days), median (IQR)* | | 296.25 (210.25–399.00) | | 285.50 (193.00–393.00) | | 0.24 |  |
| 1 HPV test, n (%) | | 172 (28.48%) | | 82 (30.59%) | |  |  |
| 2 HPV test, n (%) | | 131 (21.69%) | | 76 (28.36%) | |  |  |
| 3 HPV test, n (%) | | 109 (18.04%) | | 45 (16.79%) | |  |  |
| 4 HPV test, n (%) | | 78 (12.91%) | | 32 (11.94%) | |  |  |
| ≥5 HPV test, n (%) | | 114 (18.88%) | | 33 (12.32%) | | 0.43 |  |
| Note: *The interval between consecutive post-operative HPV tests was defined as the time interval between two adjacent post-operative HPV tests in the same patient, and was summarized using the median interval for each patient; only patients who completed at least two post-operative HPV tests were included. | | | | | | |  |
|  |  |  |  |  |  |  |  |
| **STable 7.** Observed time-specific contrasts in post-operative HPV positivity at 12, 24, and 36 months by baseline infection multiplicity | | | | | | |  |
|  |  |  |  |  |  |  |  |
| Time point | Single infection, n/N (%) | | Multiple infection, n/N (%) | | OR (95% CI)* | |  |
| 12 months | 97/433 (22.55%) | | 78/175 (44.72%) | | 2.78 (1.76–4.38) | |  |
| 24 months | 68/302 (22.70%) | | 60/130 (46.77%) | | 2.99 (1.63–5.48) | |  |
| 36 months | 44/189 (23.66%) | | 39/77 (51.16%) | | 3.38 (1.64–6.95) | |  |
| * Odds ratios compare the odds of HPV positivity in the multiple-infection group versus the single-infection group at each selected time point. For each patient, the HPV result from the closest post-operative visit within ±90 days of the target time point (12, 24, or 36 months) was used. Because this is a descriptive comparison based on observed follow-up data rather than formal GAMM-based prediction, denominators vary across time points according to the availability of HPV results within the predefined window. | | | | | | |  |
|  |  |  |  |  |  |  |  |
|  |  |  |  |  |  |  |  |
|  |  |  |  |  |  |  |  |
|  |  |  |  |  |  |  |  |
|  |  |  |  |  |  |  |  |
|  |  |  |  |  |  |  |  |

| **STable 8**. Group-specific cumulative incidence of persistence/recurrence by baseline infection multiplicity | | | |  |
| --- | --- | --- | --- | --- |
|  |  |  |  |  |
| **Outcome** | **Single infection** | **Multiple infection** | **P value** |  |
| Persistence/recurrence, n/N (%) | **106**/604 (17.55%) | 78/268 (29.10%) | <0.001 |  |
| **95% CI** | **13.52%–19.41%** | **21.86%–32.15%** |  |  |
|  | | | |  |
|  |  |  |  |  |
|  |  |  |  |  |
